# Impact of Perceived Skillset and Organizational Traits on Digital Wellbeing of Teachers: Mediating Role of Resilience

**DOI:** 10.3389/fpsyg.2022.923386

**Published:** 2022-06-02

**Authors:** Fang Yu, Farhan Mirza, Naveed Iqbal Chaudhary, Rida Arshad, Yingyu Wu

**Affiliations:** ^1^School of Economics and Management, Southeast University, Nanjing, China; ^2^Knowledge Unit of Systems and Technology (KUST) Department, University of Management and Technology, Sialkot, Pakistan; ^3^Department of Business Administration, University of the Punjab, Lahore, Pakistan

**Keywords:** perceived skillset, organizational traits, employee resilience, organizational resilience, team resilience, teachers' digital wellbeing

## Abstract

Given the current unstable and unpredictable circumstances, especially due to the COVID-19 education system has evolved, requiring frequently distinct skills, and coping behavior. This study intended to empirically test the impact of perceived skillset and organizational traits on teachers' digital wellbeing with the mediating role of three levels of resilience. To serve the cause, non-probability convenience sampling was chosen, and data was gathered through an online survey from 336 on-duty teachers in the education sector of Pakistan. The results of the study have been drawn by using the PLS-SEM partial least squares structural equation modeling technique through the Smart-PLS software 3.0 version. The findings show that perceived skillset had a positive and significant impact on digital wellbeing and organizational traits had an insignificant effect on digital wellbeing. Moreover, results indicate that organizational resilience and employee resilience positively mediate the relationship between perceived skillset and organizational traits on digital wellbeing. Similarly, findings illustrate that team resilience positively mediates the relationship between perceived skillset and digital wellbeing. Furthermore, results show that team resilience negatively and insignificantly mediates the relationship between organizational traits and digital wellbeing. Lastly, discussion, theoretical and practical implications were also discussed in this research article.

## Introduction

Driven by the technological transformation, it has become more crucial for an organization to envision an optimal way to be least affected by the precarious employment conditions already prevailing in Pakistan (Khan and Khan, 2019). Since technological developments changed the working perspective, it requires a shift in the education sector too, in terms of curriculum and teaching practices (Frank et al., 2019). During the COVID-19 pandemic technology has been widely used in Pakistan and all over the world (Lai and Bower, 2020). Yet another aspect of tech-based learning which has been ignored is the digital wellbeing of teachers because technology has made them available and accessible for 24 h which makes them feel overburdened and stressed (La Velle et al., 2020). Responding to these issues, research in the last years has focused on exploring the relationship between perceived skill set and wellbeing in education sectors and the effects of technology on teachers learning and academic achievement (Rahi, 2019), yielding contentious results; some studies emphasize the opportunities for organizational traits to pursue challenges and activities valuable for their healthy development (Bustinza et al., 2019; Shehzadi et al., 2020). In response to the COVID-19 outbreak, 57 countries in the world fully closed their educational institutes and moved to e-learning, while 95 countries partially opened with limitations (Statista., 2021). Teachers are more exposed to technology, most likely suffering from stress and anxiety, due to high expectations to fulfill the needs of each student.

Resilience refers to a person's ability to withstand or adaptively recover from stressors (Hartmann et al., 2020). Teacher resilience is defined as a complex and dynamic process which enables nurses to positively adapt to workplace stressors, avoid psychological harm and continue to provide high-quality patient care. Moreover, where operations have been transformed, the communication, collaboration, and resilient capabilities of an individual and a team are much more needed than ever (Buzzanell, 2018). COVID-19 has set a new landscape for education delivery both for students and teachers in Pakistan (Abid et al., 2021), creating the opportunity to think about technological transformation. The activities have been modified with improved digital technology and remote work, but these modifications are bringing a slew of unanticipated surprises to the forefront. The COVID-19 pandemic brought fear and uncertainty over health, safety, finances, and jobs along with longer periods of social distancing and casual lockdowns. Teachers experiencing cognitive load, poor work-life balance, and continued exposure to screens have led to increasing burnout at the workplace (Hartwig et al., 2020).

According to an estimate (Eric Patterson) American citizens spend around $300 billion, the UK $37 billion, and Australian citizens $14.2 billion on stress-related health problems. The wellbeing issues arise out of the overuse of technology especially during a lockdown, with reduced social interactions and digital overload. Teachers' and other employees' digital wellbeing have been overlooked while proposing the Digital Pakistan Policy, which is intended to digitalize almost all the departments to increase the mobility of its operations and provide the public with maximum economic wellbeing by using technology. Teaching is one of the most adopted professions in Pakistan; the number of teachers is increasing over time. During 2018–19 estimated number of teachers in public and private institutes was 1.83 million as compared to 1.77 million during 2017–18 (Iqbal et al., 2022). As it is an immensely growing field, therefore, administrators highly need to pay the attention to its corresponding policies, being more concerted to the wellbeing of teachers. During the COVID-19 pandemic, (Abu Hasan et al., 2022) reported on the rising number of instructors being employed in public and private institutes at all levels, and how the stress rate during lockdown was dangerously high among those teachers, at 89 percent. With such a large workforce in the department, it must be handled immediately by guaranteeing the best possible use of technology to avoid more economic disasters.

Existing studies on teachers' wellbeing in the digital context less empirically validate this concept and there is a scarcity of contributions by researchers in this regard (Özü et al., 2017; Vargo et al., 2021). Most of the previous studies were conducted in the context of the Western economy using qualitative research data (Cooper et al., 2019). Therefore, to fulfill this research gap this study contributes to the literature of Nyaupane et al. (2020) by empirically testing the role of perceived skillset and organizational adaptive traits on an employee, organizational, team resilience, and teacher's digital wellbeing. In addition, Kumar Pradhan et al. (2021) signified the relationship between self-efficacy and employee wellbeing as well as employee resilience which moderated the relationship between employee self-efficacy and wellbeing and suggested contributing to the research by identifying further variables that could be affecting the given relationship. Therefore, to fill this research gap this study proposed the following research questions:

RQ1: What is the impact of perceived skillset on the digital wellbeing of teachers?
RQ2: Does organizational resilience, employee resilience, and team resilience mediate the relationship between perceived skillset and teachers' digital wellbeing?
RQ3: What is the effect of organizational traits on teachers' digital wellbeing?
RQ4: Does organizational resilience, employee resilience, and team resilience mediate the relationship between organizational traits and teachers' digital wellbeing?

After the introduction section, this study is further organized into a section of critical literature review on the variables under study, research methodology, results, and conclusion of this study which provides detailed insights about the study.

## Theoretical Support and Hypotheses Development

### Resilient Leadership Theory

The resilient leadership theory is a navigating model for organizations to withstand unpredictable events and circumstances by improvising an optimistic approach among individuals and teams (Shelton et al., 2021). Prior researchers claim that resilience is one of the core traits that every leader should hold, as resilience leadership plays a vital role in shaping the follower's behavior when confronted with transition, failure, and difficult situations (Giustiniano et al., 2018). However, the resilient leadership theory is not limited to simply leaders or managers; it is a holistic process that begins at the personal level and progresses to the team, then the organization. Resilient leadership paves ways to evolve stress and anxiety into positive energy to be resilient and outperform in unfavorable circumstances through its directive competence (Rego et al., 2021). Despite the existence of resilient leadership, certain qualities may dominate the outcomes which include the organizational culture, flexibility to addictiveness, and employees' competencies (Giustiniano et al., 2018). The remote work and its consequences with regards to stress and digital overload have dramatically swelled the importance of resilient leadership to increase adaptability at all levels (individual, team, and organizational) and as a result, employee psychological stress and load can be reduced.

### Perceived Skillset and Teachers' Digital Wellbeing

Prior literature suggests that digital literacy is one of the core competencies of employees that contribute to digital wellbeing of employees (Akkermans et al., 2013). Career competencies are personal resources that employees perceive about their professional abilities, technical, communication, and critical thinking skills to initiate a motivating process. To clarify, personal resources include self-esteem, optimism, self-efficacy and are linked to resilience and one's ability to manage the environment (Hobfoll et al., 2015). Individuals are motivated to extend their stock of resources in order to safeguard and enhance their wellbeing, which improves their personal growth and development (Demerouti et al., 2019).

Furthermore, Xanthopoulou et al. (2009) found that personal resources are linked to job-related efficacy and optimism, and therefore to work engagement and reduced weariness. Plomp et al. (2019) look at how proactive workers affect their wellbeing at work and their findings imply that proactive employees may improve their wellbeing through proactive job redesign and the development of career-related skills and talents. They also discovered that career competencies are personal resources that are linked to improving self-concept and employee resilience, which are linked to better wellbeing. However, the previous literature has supported the phenomena of employees' perceived skillset affecting the wellbeing of the employees; besides it lacks empirical evidence on teachers' digital wellbeing. Therefore, this study intended to make some fruitful and novel contributions by proposing the following hypothesis;

**H1:** Perceived skillset has a positive and significant impact on teachers' digital wellbeing.

### Organizational Traits and Teachers' Digital Wellbeing

There is a major role of resilience in the prevention and diluting of the potential consequences arising out of job burnout and stress, which gave hype to the literature on organizational resilience and organizations' adaptive capacity (Kirmayer et al., 2011; Kelly et al., 2019; Salehi and Veitch, 2020). A recent study by Gardner (2020) has certain empirical evidence that by providing employees with job autonomy, experience a sense of success and similarly high resilience and adaptive behavior, which result in their high wellbeing.

According to the organizational support theory (OST), employees who perceive their organizations as valuing their contributions and caring about their wellbeing will reciprocate with favorable attitudes and desirable behavior toward their organizations (Eisenberger et al., 1986; Rockstuhl et al., 2020). This win-win relationship has gained considerable academic attention since the groundbreaking work of Eisenberger et al. (1986), with abundant research highlighting the favorable outcomes of perceived organizational support including job satisfaction, job embeddedness, organizational commitment, and organization-based self-esteem.

Moreover, Wattoo et al. (2018) found that perceived organizational support has a positive and significant impact on tour guides' quality of work-life, while also having a negative and significant impact on their levels of burnout. Kurtessis et al. (2017) noted the paucity of quantitative research that addressing on how an adaptive organization can play its role in the digital wellbeing of its employees. Thus, reflecting the novelty of this study, the following hypothesis has been formulated;

**H2:** Organizational traits have a significant impact on teachers' digital wellbeing.

### Mediating Effect of Organizational Resilience

Thompson and Dobbins (2018) argue that organizational resilience is a complex system that impacts the environment, society, and employee responses toward certain events, particularly the person's life. Several studies support the phenomena that an organizations' supportive and learning culture contributes to employee engagement, resilience, and subjective satisfaction (Mazzetti et al., 2016; Meintjes and Hofmeyr, 2018). Moreover, the knowledgeable employees perceive themselves as more secure which indulge greater job satisfaction and a sense of commitment or relatedness to the organization in turn to organizational resilience capacity (Al-Omar et al., 2019).

Moreover, Prayag et al. (2020) measured employee wellbeing in terms of satisfaction, providing empirical evidence that the organization's provision of resources, flexible environment, adaptive traits, and supportive environment contribute to their subjective wellbeing. Previous studies support this relationship by comprehending that if organizations are being supportive, adaptive, readily accept and respond to change, an individual's capability to cope with stress and unpleasant events will be enhanced (Seville, 2018; Tonkin et al., 2018; Näswall et al., 2019). Furthermore, prior literature signifies a relationship between organizational learning or adaptive performance and employees' ability to cope with uncertainties and unpredictable environments (Pradhan et al., 2017). Therefore, it can be expected that organizational resilience mediates the relation between employees' perceived skillset and digital wellbeing. Hence, we propose the following hypothesis;

**H3:** Organizational resilience mediates the relationship between perceived skillset and teacher's digital wellbeing.
**H4:** Organizational resilience mediates the relationship between organizational traits and teachers' digital wellbeing.

### Mediating Role of Employee Resilience

Prior studies on resilience show that employees' capabilities contribute to their adaptation and coping skills during traumatic and astonishing situations which allow them to rapidly recover from the crisis they confront, while their response to the crises is driven by their perception, past experiences, and culture (Thompson and Dobbins, 2018; Brassington and Lomas, 2021). Employees' wellbeing and their resilience capabilities have a direct relationship according to prior studies (Gardner, 2020). Moreover, scholars argue that individuals' resiliency decreases anxiety and improves satisfaction and happiness (Babanataj et al., 2019; Cooper, 2019). There is a major role of organizational context, culture, and type of conduct in the employee is involved are considered in developing resiliency.

Furthermore, prior studies claim that organizational processes and practices contribute to the knowledge and capacity of its employees which in turn add up to the ability of an organization to cope with crisis (Prayag et al., 2018; Chowdhury et al., 2019). In addition to this, individual traits significantly impact organizational outcomes in terms of performance (Zhang and Acs, 2018). Additionally, literature discloses that knowledge creation by an organization by multiple means and the readiness of its employees are contingent on its adaptability to tempestuous and unpleasant situations which in turn encourages employee resilience and decreases stress levels (Hussein et al., 2016; Pradhan et al., 2017). The empirical findings support the relationship between learning organizational culture and subsequent employee engagement and resilience significantly (Cooke et al., 2019a; Malik and Garg, 2020). Thus, this study assumes that to achieve the wellbeing of teachers, optimizing resilience at the individual level can play a huge role. Thus, this study predicted the following hypotheses;

**H5:** Employee level resilience mediates the relationship between perceived skillset and teacher's digital wellbeing.
**H6:** Employee level resilience mediates the relationship between organizational traits and teachers' digital wellbeing.

### Mediating Role of Team Resilience

The resilience concept is concerned with an individual or community's abolition to adapt to the economic, industrial, or environmental changes and make transformations, respectively (Søholt et al., 2018). Hartmann et al. (2021) provide empirical evidence by indicating how emotional culture and happiness are important for mutual relationship goals, and reflexivity that in turn enhances team resilience, and the team's positive psychological capacity and organizational behavior are also considered to play a vital role. The prior study revealed a significant relationship between team resilience and organization effectiveness, and it positively impacts the competitive advantage (Sharma and Sharma, 2020). Team resilience can provide the basis for the development of resilient capabilities at the individual level to cope the stressful events (Hartwig et al., 2020). However, the literature lacks empirical evidence on how team resilience can be impacted and contributes to the prevalence of wellness (Varajao et al., 2021). Thus, the following hypotheses are proposed;

**H7:** Team-level resilience mediates the relationship between perceived skillset and teachers' digital wellbeing.
**H8:** Team-level resilience mediates the relationship between organizational traits and teachers' digital wellbeing.

### Conceptual Model

**Figure 1:**
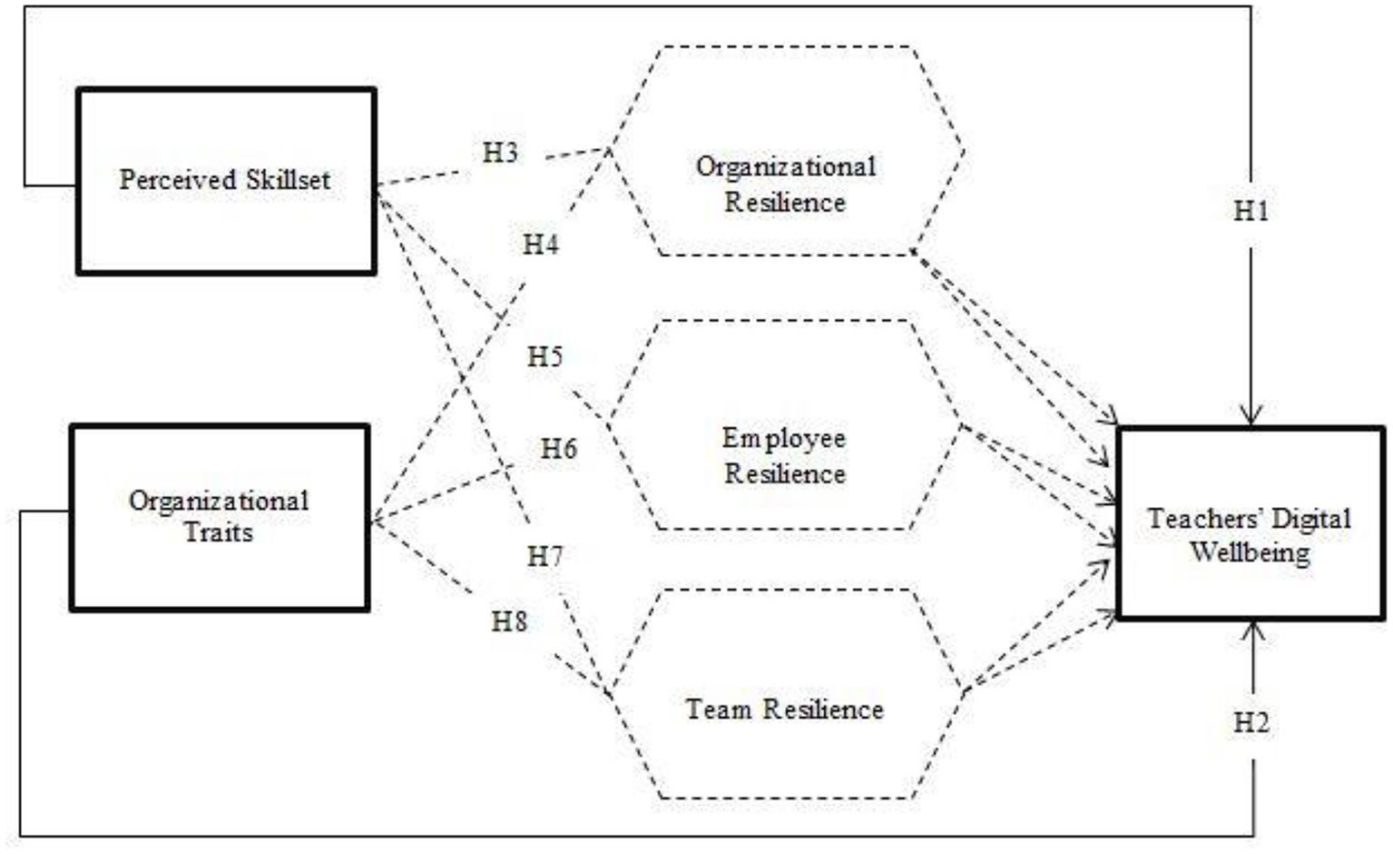
Conceptual model.

The conceptual model depicting the relationships and hypothesis is given in Figure 1.

## Methods

### Research Approach and Sampling Technique

This research followed a survey strategy related to the deductive approach. The strategy has gained popularity as it allows an innumerable amount of data collection in a highly economical way from a diverse pool of participants (Saunders et al., 2018). This study context was the education sector of Pakistan, as the evidence shows the higher rate of teachers were employed in the country, i.e., during 2018–19 estimated number of teachers was 1.83 million. However, it was ensured that these teachers are interacting with students through digital sources during the time of the COVID-19 pandemic, to address the problem of digital wellness. Therefore, the population of the given study included teachers from Pakistan.

According to Sekaran and Bougie (2016) for an unknown population size, the sample size from 50 to 700 respondents is sufficient for most social and behavioral science studies.

According to Mason (2010), the formula for calculating the sample size *z*^2^ × *p* (1 – *p*)/e^2^, Where *z* = 1.6384, *p* = 0.25, and *e*^2^ = 0.0016, the sample size for this study is approximately 600. Therefore, the authors distributed 570 questionnaires to the teachers of educational institutions in Pakistan. Since it was impossible to get accurate information about the total number of teachers, working in educational institutions in Pakistan, the authors preferred to follow a convenience-based sampling technique to collect the data. The given sampling technique has also been considered an ideal approach when we do not have access to the whole population.

### Data Collection Method

The current research followed an empirical approach to collect the data. Data were collected through a structured questionnaire using a Web-based platform, i.e., Google form during the period of COVID-19. To endure impartiality in responses, a cover letter was attached with a questionnaire that explained the purpose of the research and ensured respondents that their responses would remain anonymous and their participation in this study was completely voluntary. The self-administered questionnaires were distributed through email to 570 respondents (due to COVID-19, it was difficult to approach the employees individually, and outsiders were also not allowed to enter the company). First hand, only 245 filled questionnaires were returned after 3 weeks. To collect the data from the remaining respondents, the soft reminder calls through email were made again, and 124 more responses were received effectively. After removing unusable questionnaires (excluded during the data screening process due to incomplete or invalid responses), 336 useable questionnaires were obtained.

### Measures

The items were selected from validated questionnaires used in previous research studies and presented in English as it is the official language of educational institutions in Pakistan. The scaling technique was used to measure the responses, i.e., a five-point Likert scale (1 = strongly disagree to 5 strongly agree) for all scales. The questionnaire consisted of seven parts: the first part contained information about the participants' demographics, followed by measures for perceived skillset, teacher's wellbeing, organizational traits, organizational resilience, employee resilience, and team resilience. Moreover, the survey scales used in this study comprised 56 questions relating to six constructs and four questions about respondents' demographic variables.

#### Teachers' Digital Wellbeing

The digital wellbeing of teachers was measured with the 14 items that accurately demonstrate the characteristics of physical, psychological, and social wellbeing dimensions, by adapting the scale of Passey (2021). However, in our study, we adapted the scale by including closed-ended questions and excluding 2 open-ended items that index the physical, psychological, and social wellbeing of teachers while incorporating digital technology into their job. A sample item includes “digital technologies offer more opportunity to work independently”.

#### Perceived Skillset

Perceived skillset contained perception of employees about their occupational skills, technical, communication, and critical thinking skills. The scale was consisted of 11 items and adapted from prior published studies (Mallak, 1998; Gilley et al., 2008; Smith and Stirling, 2010; Robles, 2012). Moreover, Nyaupane et al. (2020) validated the scale with (Cronbach's α = 0.85). The sample item for the scale was “I am confident about my occupation-specific skills”.

#### Organizational Traits

Organizational traits were measured using the flexibility of operations, organizational environment, shared goals, and sense of purpose. The 14 items scale was adapted from prior studies (Lengnick-Hall et al., 2011; Lee et al., 2013). Furthermore, Nyaupane et al. (2020) validated the scale by reporting the value of Cronbach α = 0.93. A sample item includes “My institute provides a safe, trusting work environment”.

#### Employee Resilience

To measure employee level resilience, the adopted scale contains 5 items that were developed and validated by Caza and Bagozzi (2010). Employee resilience contained the compatibility of an employee at the workplace when he confronts some unpleasant situations. Stephens et al. (2013) validated the scale items with a reported value of Cronbach's α = 0.87. A sample question includes “I resolve crises competently at work.”

#### Organizational Resilience

Organizational resilience was measured by using 5 items scale developed and validated by Lee et al. (2013). This scale was further validated by Orchiston et al. (2016), and the reported value of Cronbach's α = 0.89. Moreover, Prayag et al. (2020) also adapted the same scale in their study. A sample item is “Our institute maintains sufficient resources to absorb some unexpected changes.”

#### Team Resilience

The 4 items scale was adapted to measure team-level resilience. This scale was developed by Mallak (1998). Sample item includes “In a team, individuals have a shared understanding of the team's mission and can fill in wherever needed to ensure smooth functioning of the team.”

### Measurement Model Analysis

To assess content and face validity, the initial questionnaire was reviewed by academic experts who specialized in the areas of human resource management and specifically the leadership domain before distributing the link among the respondents. They thoroughly reviewed the content of the questionnaire and the extent to which it was likely to measure the study variables. However, positive comments were received from all the experts. Therefore, no further changes were introduced to the instrument used for the pilot study, and the same was carried forwards for the comprehensive study. Before conducting the comprehensive survey, as recommended by Hinkin (1998), the authors performed a pilot study to test the questionnaires' feasibility, clarity, appropriateness of the questionnaires. Therefore, the pre-tests were designed and developed to ensure that the measures were logically consistent, complete, and valid. The internal reliability of the study and the research instrument for the pilot study was measured through Cronbach's Alpha (α). The pilot test among 45 respondents revealed that Cronbach's alpha for all the constructs exceeded the acceptable range of 0.70. These figures showed that the scales used in this research are consistent and reliable. Additionally, the pilot study results suggested that the proposed questionnaires instrument is understandable, clear, and can be answered in around 7–8 min.

### Data Analysis Technique

By using SPSS, demographics, descriptive statistics, and tests of normality were determined. Smart PLS version 3.0 was used for confirmatory factor analysis, internal accuracy and validity estimates, hypothesis checking, and mediation testing. Depending on different factors, we applied the PLS-SEM method to evaluate our hypotheses. Prior researchers claim that PLS avoids many of the restrictive assumptions underpinning maximum likelihood techniques and protects against inaccurate solutions and factors indeterminacy (Almazroi et al., 2018; Ahmad et al., 2021). PLS-SEM has no distributional assumptions on the error terms and PLS can handle both reflecting and formative constructs. PLS is a latent variable modeling approach that includes many dependent constructs and explicitly identifies measurement error (Schwarzer et al., 2021). Moreover, unlike covariance bases SEM techniques, PLS is unaffected by sample size constraints and is appropriate for any sample size greater than thirty. Our sample consists of 336 respondents, thus, we have a sample that requires PLS-SEM (Veronese et al., 2018).

## Results

### Data Analysis Technique

Before analyzing the data, it is necessary to check some statistical values for the normal distribution of data. For conducting parametric tests such as ANOVA, regression, and structural equation models, the distribution of data should be normal. Skewness and kurtosis values must be between +1 and −1 (Groeneveld and Meeden, 1984; Byrne, 2010). As seen in Table 1 descriptive statistics, skewness, and kurtosis values are between +1 and −1. Therefore, we apply SEM as one of the parametric analyses.

**Table 1:** Descriptive statistics.

**Variables**	**Mean**	**Std. deviation**	**Skewness**		**Kurtosis**	
	**Statistic**	**Statistic**	**Statistic**	**Std. error**	**Statistic**	**Std. error**
PS	3.4997	1.09360	−0.736	0.133	−0.905	0.265
OT	3.4598	1.08088	−0.647	0.133	−1.079	0.265
OR	3.4464	1.12777	−0.646	0.133	−1.038	0.265
TR	3.2253	0.98937	−0.058	0.133	−0.695	0.265
ER	3.5006	1.09403	−0.771	0.133	−0.853	0.265
DW	3.4318	1.05378	−0.634	0.133	−0.478	0.265

*PS, Perceived skillset; OT, Organizational traits; OR, Organizational resilience; TR, Team resilience; ER, Employee resilience; DW, Digital wellbeing*.

### Measurement Model Analysis

The composite scale reliability (CR), Cronbach's alpha (CA), and average variance extracted (AVE) were used to assess reliability. For all constructs, PLS-based CR is above the threshold value of 0.70, Cronbach's alpha CA exceeds the threshold value of 0.70, and AVE surpassed the threshold value of 0.50 (Hair et al., 2010). In addition, we assessed convergent validity by looking at the standardized loadings of the measures on their respective constructs, and we discovered that all of them exceed 0.70 (Bagozzi and Yi, 1988). Moreover, Table 2 presents the correlation between all six constructs, demonstrating discriminant validity. AVE for each dimension should be predicted to be higher than the squared correlation between components to completely meet the standards for discriminant validity (Fornell and Larcker, 1981). None of the constructs' inter-correlations surpassed the square root of the AVE of the constructs in the model. Furthermore, we assessed convergent validity by examining the standardized loadings of the measures on their respective constructs, and we discovered that all of the measures have standardized loadings >0.70.

**Table 2:** Reliability, validity, and correlations.

**Constructs**	**DW**	**ER**	**OR**	**OT**	**PS**	**TR**
DW	0.856					
ER	0.426	0.873				
OR	0.411	0.410	0.877			
OT	0.296	0.556	0.571	0.836		
PS	0.490	0.576	0.522	0.512	0.856	
TR	0.589	0.316	0.250	0.164	0.409	0.861
**Reliability and validity**						
CR	0.925	0.941	0.943	0.970	0.968	0.920
AVE	0.732	0.762	0.769	0.699	0.733	0.741
CA	0.927	0.922	0.925	0.967	0.963	0.883

*PS, Perceived skillset; OT, Organizational traits; OR, Organizational resilience; TR, Team resilience; ER, Employee resilience; DW, Digital wellbeing; CR, Composite reliability; AVE, Average variance extracted; CA, Cronbach's alpha*.

### Structural Model

**Figure 2:**
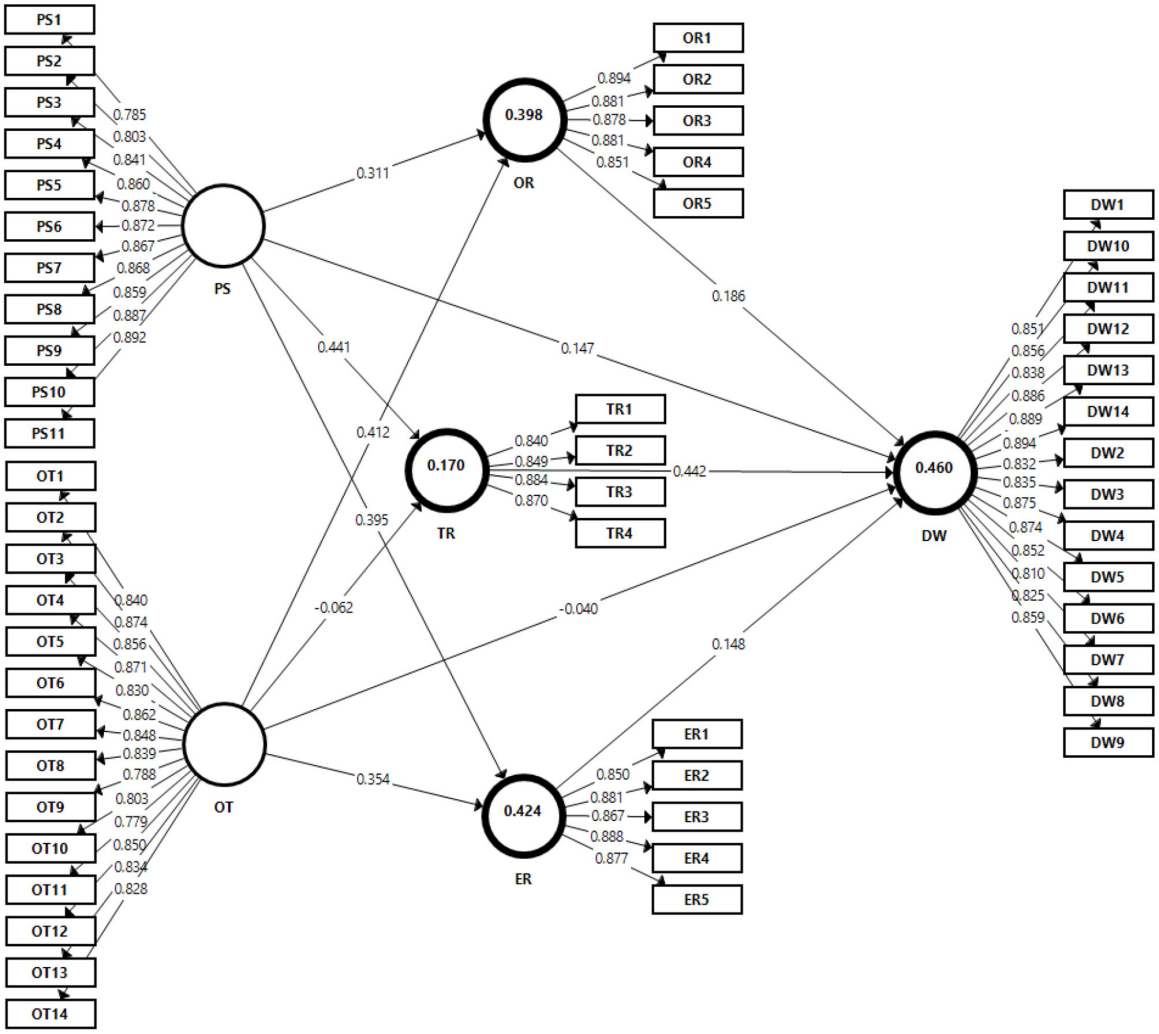
Structural model.

Various quality scores, such as the coefficient of determination (*R*^2^), predictive validity (*Q*^2^), and SRMR are used to verify the PLS-SEM technique. The values of *R*^2^, *Q*^2^, and SMRM were shown in Table 3 and Figure 2. The endogenous constructs *R*^2^-values are used to assess model fit and determine how well data points match a line or curve. According to Chin (1998) *R*^2^ levels can be classified as small (0.02 < *R*^2^ < 0.13), medium (0.13 < *R*^2^ < 0.26), or large (0.26 < *R*^2^). The endogenous constructs' R^2^ statistic values were utilized to test model fit (Sarstedt et al., 2014). The values of organizational resilience (*R*^2^ = 0.398); team resilience (*R*^2^ = 0.170); employee resilience (*R*^2^ = 0.424) and digital wellbeing (*R*^2^ = 0.460). All the constructs *R*^2^ have large effect sizes. All of our endogenous constructs' *Q*^2^ validity was similarly satisfactory. This result indicates that *Q*^2^ results were also satisfactory and explain the variation in the dependent variable. Moreover, SRMR (standardized root mean squared residual) should be equal to or <0.08 (Henseler et al., 2014), and results indicate that SRMR for our model is 0.045, which meets this criterion.

**Table 3:** Structural model.

**Endogenous variables**	***R* ^2^**	***Q*^2^ (=1-SSE/SSO)**
OR	0.398	0.301
TR	0.170	0.123
ER	0.424	0.318
DW	0.460	0.330

*OR, Organizational resilience; TR, Team resilience; ER, Employee resilience; DW, Digital wellbeing*.

#### Hypothesis Testing

##### Direct Effects

**Figure 3:**
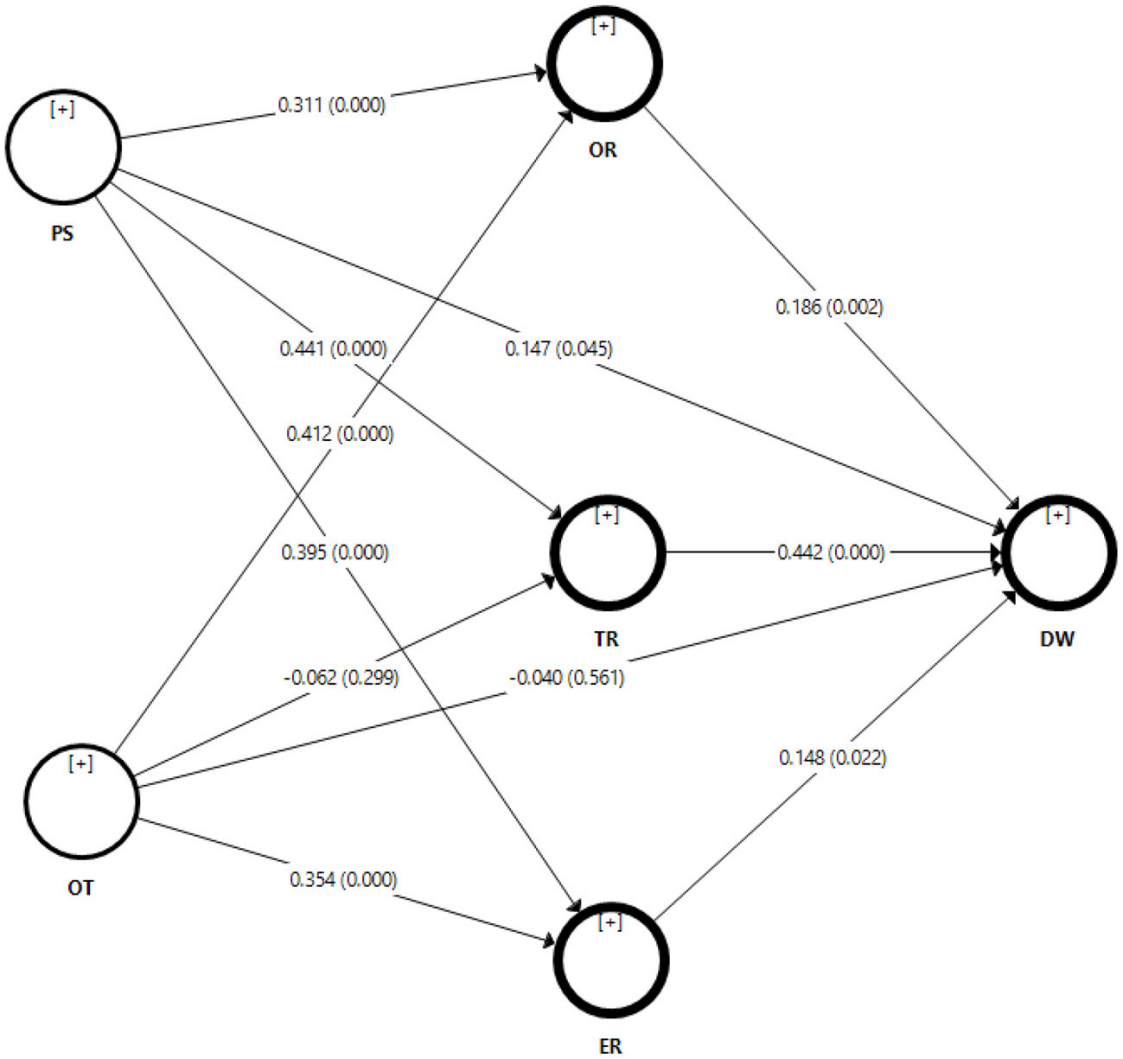
Bootstrapping.

The hypothesis relationships were tested through PLS-SEM partial least squares structural equation modeling technique. The results were presented in Table 4 and Figure 3. All the results were statistically significant to expect (H2 and H8). To test hypothesis H1 results show that perceived skillset had a positive and significant impact on digital wellbeing (β = 0.147; *t* = 2.007; *p* < 0.045). Therefore, H1 was accepted. Meanwhile, H2 findings indicate that organizational traits have a negative and insignificant influence on digital wellbeing (β = −0.040; *t* = 0.581; *p* < 0.561). So, H2 was not accepted.

**Table 4:** Direct relationships.

**Hypotheses**	**Relationships**	**β**	***t***	***p***
H1	PS → DW	0.147	2.007	0.045
H2	OT → DW	−0.040	0.581	0.561
-	ER → DW	0.148	2.296	0.022
-	OR → DW	0.186	3.029	0.002
-	OT → ER	0.354	5.992	0.000
-	OT → OR	0.412	6.062	0.000
-	OT → TR	−0.062	1.039	0.299
-	PS → ER	0.395	6.771	0.000
-	PS → OR	0.311	4.499	0.000
-	PS → TR	0.441	8.138	0.000
-	TR → DW	0.442	9.216	0.000

*PS, Perceived skillset; OT, Organizational traits; OR, Organizational resilience; TR, Team resilience; ER, Employee resilience; DW, Digital wellbeing*.

##### Indirect Effects

To test the mediation effects of organizational, team, and employee resilience in the relationship between perceived skillset and organizational traits on digital wellbeing. We applied () mediation approach to examine the indirect effects using the 97.5% confidence intervals method. The findings were shown in Table 5. H3 results illustrate that organizational resilience positively mediates the relationship between perceived skillset and digital wellbeing (β = 0.058; *t* = 2.473; *p* < 0.013). Hence, H3 was supported. Similarly, H4 findings show that organizational resilience positively mediates the relationship between organizational traits and digital wellbeing (β = 0.077; *t* = 2.758; *p* < 0.006). Thus, H4 was accepted. Furthermore, H5 findings indicate that employee resilience positively mediates the relationship between perceived skillset and digital wellbeing (β = 0.058; *t* = 2.148; *p* < 0.032). Therefore, H5 was supported. Additionally, H6 results explain that employee resilience positively mediates the relationship between organizational traits and digital wellbeing (β = 0.052; *t* = 2.090; *p* < 0.037). Consequently, H6 was accepted. Besides, H7 results show that team resilience positively mediates the relationship between perceived skillset and digital wellbeing (β = 0.195; *t* = 6.488; *p* < 0.000). Thus, H7 was supported. Lastly, H8 findings indicate that team resilience had a negative and insignificant mediating effect on the relationship between organizational traits and digital wellbeing (β = 0.077; *t* = 2.758; *p* < 0.006). Therefore, H8 was not supported.

**Table 5:** Indirect relationships and confidence intervals.

**Hypotheses**	**Relationships**	**β**	***t***	**2.5%**	**97.5%**	***p***	**Mediation results**
H3	PS → OR → DW	0.058	2.473	0.018	0.109	0.013	Partial mediation
H4	OT → OR → DW	0.077	2.758	0.027	0.136	0.006	Partial mediation
H5	PS → ER → DW	0.058	2.148	0.010	0.116	0.032	Partial mediation
H6	OT → ER → DW	0.052	2.090	0.008	0.105	0.037	Partial mediation
H7	PS → TR → DW	0.195	6.488	0.139	0.256	0.000	Partial mediation
H8	OT → TR → DW	−0.027	1.041	−0.079	0.024	0.298	No mediation

*PS, Perceived skillset; OT, Organizational traits; OR, Organizational resilience; TR, Team resilience; ER, Employee resilience; DW, Digital wellbeing*.

## Discussion

The remote-education has caused multiple opportunities as well as challenges for the teachers at a professional level, such as maintaining work-life balance while working from home and digital interaction which impacts their physical movement, increases psychological tension and social connections. In this study, we empirically tested how perceived skillset and organizational trails are associated with the digital wellbeing of teachers in Pakistan. To summarize, we tested eight hypotheses and two were not supported. The findings support employees' perceptions about their skillset directly affecting the digital wellbeing of teachers (H1), in accordance with the current circumstances where employees perform inside organization boundaries, during COVID-19. This significant relationship could provide the higher and different work demands, role conflict, the poor work-life balance that has arisen due to increased digital workload and working from home. In addition to this, it decreases their social interaction, they are physically at home but mentally in the office for 24 h, also their productivity is interrupted by their family, all these factors increase the cognitive load on an employee. Prior researchers argue that the perceived skillset of teachers and professional learning play a huge role to prepare them to face uncertain challenges and cope with stress and workload (Clarà, 2017). Moreover, an existing study found that contextual elements have a greater impact on subjective wellbeing, satisfaction, stress, and burnout as compared to perceptual components (Ainsworth and Oldfield, 2019). Furthermore, previous researchers provide an insignificant relationship between perceived skillset and digital wellbeing (Fernandes et al., 2019). Therefore, this study contributes to the significant impact of perceived skillset on digital wellbeing.

Against the proposed hypothesis that organizational traits significantly impact the digital wellbeing of teachers (H2), it has been observed by analysis that organizational traits do not directly enhance or reduce digital wellbeing. A prior study conducted on the service sector employees in Malaysia also did not find the following relationship significant (Johari et al., 2019) due to contextual and situational differences, and provides supportive grounds to our findings. Gardner (2020) provides certain empirical evidence that the provision of job autonomy to employees at the workplace develops a sense of success and similarly high resilience, while digital wellbeing is a new criterion in this case. The supportive school's activities reduce anxiety and stress among staff. However, considering the current circumstances of social distancing and conducts shifting to digital platforms, one of the crucial issues was digital access, as only 15% of teachers had digital access and due to this sudden shift, they were not provided proper training on digital literacy, which restricted their level of performance and consequently, increased their increased burnout. Besides contextual factors, students' distracting behavior with the use of technology, impacts the resilience and wellbeing of teachers, they tend to prefer face-to-face learning instead of online classes (Aguilera-Hermida, 2020). The lack of proper assistance from teachers and learning level is low which is unconformable for both teachers and students as the teacher's satisfaction is ultimately associated with students' satisfactory responses and behavior (Hussein et al., 2020; Shehzadi et al., 2020).

Regarding the mediation effects of three levels of resilience; organization, individual, and team level resilience, this study is the first one that investigated such mediation effects on the relationship between perceived skill, traits of organization, and digital wellbeing of teachers. Our third proposed hypothesis, organizational resilience mediates the relationship between perceived skills and digital wellbeing (H3). This hypothesis holds significant value, as organizational resilience fully mediated the relationship. Several studies support our results that organizations' supportive environment contributes to employee engagement, resilience, and subjective satisfaction (Mazzetti et al., 2016; Meintjes and Hofmeyr, 2018).

The next hypothesis of the study is that organizational resilience mediates between organizational traits and teachers' digital wellbeing (H4). Our results are aligned with a study conducted by Prayag et al. (2020) which provides evidence that the provision of resources, flexible environment, adaptivity, and supportive environment improve satisfaction or subjective wellbeing among employees. Existing studies also validated the relationship between supportive organizations and an individual's coping abilities to deal with stress, which in turn proves to be a source of wellbeing (Seville, 2018; Tonkin et al., 2018; Näswall et al., 2019).

Moreover, employee resilience mediates the relationship between perceived skillset and digital wellbeing. The hypothesis (H5) was supported. We observed partial mediation of employee-level resilience. Since this rare relationship does not exist in literature but a cross-national study that has quite similar variables revealed that personality, values, and moral foundations (the constructs which shape an individual's perceptions) have a direct relationship with wellbeing, resilience, job performance, and job satisfaction (Athota et al., 2020). Some other studies also support our results and argued that employees' wellbeing and their level of resilience capabilities have a direct relationship (Gardner, 2020; Brassington and Lomas, 2021).

Another proposed hypothesis that employee level resilience mediates the relationship between organizational adaptive traits and teachers' digital wellbeing (H6) was accepted. Our results matched with prior studies which signified the partial mediation of employee resilience between wellbeing-oriented practices at the workplace and supportive culture at an organization that enhances employee performance and subsequent satisfaction at the workplace (Cooke et al., 2019b; Cooper et al., 2019). Moreover, these studies were conducted in similar situations, as the world is witnessing the most devastating pandemic in history, and woke demands have changed.

Finally, we proposed that team resilience mediates the relationship between perceived skillset (H7) and digital wellbeing and insignificantly mediates the relationship between organizational traits and digital wellbeing (H8). The results revealed the significant and insignificant mediation of team resilience on the relationship. The literature lacks empirical evidence that team resilience has any direct or direct effects on wellbeing (Varajao et al., 2021). However, a dissimilar study provides little support to our result by empirically validating, that emotional culture and happiness are significant for mutual relationship goals, and reflexivity that in turn enhances team resilience (Hartmann et al., 2021). While teams' positive psychology plays a significant impact on the overall organizational behavior and wellness. Apart from peer support and collaborations, leadership has a crucial role in building resilience capability at all organizational levels during crisis and motivation. Although the responsibility of leaders was already increased before the forced digitization (Vial, 2019; Bartsch et al., 2021), however, their role became even more crucial during this COVID-19 pandemic. Also, the reasons for significant and insignificant mediation for organizational and team resilience could be that team resilience is achieved through shared beliefs and goals, multi-skills, networks, and joint learning orientation, creating a greater level of synergy among the teachers. This higher level of orientation and shared values and synergy guards them against unfavorable circumstances (Sharma and Sharma, 2020), and could be a great source of order to overcome their exhaustion levels.

### Practical Implications and Limitations

This study contributes to the literature by providing several avenues and insights on digital wellbeing. In particular, the present study contributes to the existing literature by linking the perceived skillset of teachers, subsequent traits of the organization they provide their services through, and the presence of three levels of resilience, contributing to their digital wellbeing. This study would help researchers specify the path to the identification of multiple components impacting teachers' wellbeing, and other employees performing in the service sector, during the age of digitalization. Researchers can develop different training modules and training mixes which will enhance digital wellness plus awareness of employees in various sectors.

Similarly, this study has provided a fair domain to the literature about the existing issues and problems faced by teachers, with regard to technology use in their profession; further research is needed to define optimal measurement strategies as a guide for digital wellbeing. The study results also provide a promising direction for policymakers. In response to the emergencies that emerged due to COVID-19, the finding of the study revealed that the government and the responsible institutes for implementing educational policies, should make efforts and contribute by utilizing research capacities to respond to the unaddressed problem of digital wellbeing of teachers at all levels and provide with sustainable solutions. To sum up, an effective digital wellbeing program should be part of education policy, which is documented, budgeted, have specific teams to implement and run it, by using data and technology as enablers.

Along with policymaking, this study has significant practical implications not only for the education sector but for other sectors whose employees are performing tasks using digital platforms. The organizations should ensure positive adaptation processes and flexible work hours, surprise breaks, and allow for mobile switch off on weekends to lessen the digital burden on employees. Due to differences in digital dexterity among teachers, a training session on digital awareness should be arranged at the institutional level to enable them with the best use of digital workplaces and a better student-teacher interaction. Furthermore, administrators make sure the availability of uninterrupted service providers to teachers, to assure quality systems of education through digital means.

This study has some limitations that would be considered in future research directions. First, the undertaken data was based on self-report measures and using the only instrument of an online questionnaire which increases the chances of common method bias. However, an e-questionnaire was incorporated to collect study data considering the current circumstances of COVID-19 and social distancing. Nevertheless, further studies can collect data by using multiple instruments, using mixed methods, i.e., quantitative and qualitative. Second, this study incorporates cross-sectional data from teachers of Pakistan to validate the significance of the relationship among variables under study; future studies can go with a longitudinal approach to explore the change patterns in digital wellbeing concerns of teachers during and after COVID-19.

Third, we only focused on the mediating effects of employee, organizational and team-based resilience to determine the effects of perceived skillset and organizational traits on the digital wellbeing of teachers in Pakistan, future researchers can contribute by additional stressors that directly or indirectly impact their wellbeing, i.e., job complexity, financial status, and digital literacy. Finally, the researchers used a quantitative approach to analyze the primary data; future research can encompass both qualitative and quantitative to extend the understanding of several contextual components related to digital concerns, and how the wellbeing of teachers can be supported by effective and responsible use of technology. Future research can further explore the topic among multi-samples or comparative-based analysis among the provinces or countries.

## Conclusion

Due to widespread recognition of wellbeing at the workplace and emergent challenges for teachers due to the use of technology, this study identified a gap in the literature and is attempted to disclose the rarely sketched issue of digital wellbeing of teachers in the education sector of Pakistan, through empirical evidence. This study implies that the perception of teachers does not necessarily impact their wellbeing, the institutes or organizations should be more focused on the developmental needs of teachers. As time requires the academic activities to shift and being more dependent on digital technologies, teachers must be equipped with the digital skills to maintain their wellbeing. Digitization has significant importance and improves learning for both teacher and student, and of course, it is the need of the hour to minimize the setbacks of COVID-19. Educational institutions need to reassess the gaps that exist in the professional skills of the teacher, as the sudden shift in academic activities did not allow teachers to respond and adapt to changes effectively. In a country like Pakistan, where most of the citizens are unaware of digital literacy, teachers are most probably feeling anxious, who were not equipped with the relevant devices and facilities to join the lane. Meanwhile, teams of teachers were framed to complete certain tasks at the school and college level in Pakistan, and during the pandemic, their roles became more vibrant.

Results have highlighted the importance of being resilient individually and as a team, giving them more satisfaction and they feel more at ease while using technology. The combined efforts, joint decision-making in teams, collaboration, and joint coping efforts significantly impact the digital wellbeing of teachers. On the other hand, developing resilient capabilities among teachers through proper professional training and digital literacy will allow teachers to bounce back to unprovoked situations. Moreover, resource availability and decision-making at the institution level are some of the elements that contribute to agility and resilience, it boosts the confidence of teachers, and they can easily cope with certain situations. Altogether study findings suggest that educational institutes should pay more attention to the skills and resiliency of teachers, which will improve their performance and coping abilities to deal with certain situations, enhance personal assets, and subsequent wellbeing.

## Data Availability Statement

The raw data supporting the conclusions of this article will be made available by the authors, without undue reservation.

## Ethics Statement

The studies involving human participants were reviewed and approved by Southeast University, Nanjing, China. The patients/participants provided their written informed consent to participate in this study.

## Author Contributions

FY, FM, and NC proposed the research, analyzed the experimental results, and wrote the manuscript. YW and RA designed and carried out the experiments and extensively edited the manuscript. All authors contributed to the article and approved the submitted version.

## Conflict of Interest

The authors declare that the research was conducted in the absence of any commercial or financial relationships that could be construed as a potential conflict of interest.

## Publisher's Note

All claims expressed in this article are solely those of the authors and do not necessarily represent those of their affiliated organizations, or those of the publisher, the editors and the reviewers. Any product that may be evaluated in this article, or claim that may be made by its manufacturer, is not guaranteed or endorsed by the publisher.
