# Supplementary material for: Impact of Perceived Skillset and Organizational Traits on Digital Wellbeing of Teachers: Mediating Role of Resilience
Source: Front Psychol. 2022 Jun 2;13:923386. doi: 10.3389/fpsyg.2022.923386 (PMC9201954; doi:10.3389/fpsyg.2022.923386)
Supplement: Supplementary file 1 [file Data_Sheet_1.pdf]

Appendix

Teachers' Digital wellbeing

1. Digital technologies offer more opportunity to work independently
2. Using the digital technology, it reduces long weekday hours
3. Using the digital technology, it reduces weekend working
4. Using the digital technology, it offers a better work/life balance
5. Using digital technology, I find I have more time to be with family and friends
6. Using digital technology, I feel more able to switch off and relax
7. Using digital technology, I find it enables me to do more physical exercise
8. Using digital technology reduces anxiety
9. Using digital technology reduces depression
10. Using digital technology reduces exhaustion
11. Using digital technology reduces stress
12. Using digital technology reduces workload
13. Using digital technology reduces reliance on ways to alleviate stress
14. Using digital technology reduces reliance on tools considered unhealthy

Perceived skill set

1. I feel comfortable using information technology and information management
2. I apply creative, innovative thinking while working
3. I ensure work ethic, attitude, and professionalism at workplace
4. I'm meeting the work goals through analysis, critical Thinking, problem solving, and reasoning
5. I feel confident about meeting my students learning demands
6. I have broad environmental sciences understanding
7. I encourage collaboration and teamwork
8. I am confident about my occupation specific skills
9. I feel at ease to communicate with my peers

10. I'm optimistic about ongoing learning
11. I have a clear cultural understanding of my workplace

Organizational traits

1. My institute provides safe, trusting work environment
2. My institute allows thinking beyond status quo
3. My institute desire to change or improve
4. I believe right people are involved in decision-making process in my institute
5. My institute has the ability to change organizational structure based on need
6. My organization provide employees with adequate Skills
7. My institute is focused effective long-term planning and thinking
8. I believe that my institute has ability to experiment
9. My institute highly admires diverse thoughts
10. In my institute shared sense of purpose is prevailed
11. My institute facilitates flexible teaching practices
12. The decision-making is based on the experiment in my institute
13. My institute is capable of generating public interest with education
14. I believe that my institute is providing adequate, genuine opportunities for public and stakeholder input

Resilience

Organizational resilience

People in our organization are committed to working on a problem until it is resolved

Our organization maintains sufficient resources to absorb some unexpected change

If key people were unavailable, there are always others who could fill their role

There would be good leadership from within our organization if we were struck by a crisis

We are known for our ability to use knowledge in novel ways

Employee resilience

I am getting better at my work because I learn from my mistakes

Dealing with difficult colleagues (or situations) enables me to grow

I see challenges as an opportunity to learn

I find ways to handle unexpected situations

I bounce back when I confront setbacks at work

Team resilience

The team perceives change as opportunity, not danger

In team we develop the ability to create solutions on the spot using materials on hand

In team we develop the ability to make decisions with less than the desired amount of information

In a team, individuals have a shared understanding of the team's mission and can fill in wherever needed to ensure smooth functioning of the team

Thank you
